# Obituary.—Dr. Daniel P. Bissell

**Published:** 1874-11

**Authors:** 


### Editorial.

### Obituary.

DANIEL P. BISSELL, of Utica, N. Y., died Wednesday October 28th, at the advanced age of seventy-two.

DR. BISSELL was widely known by the members of the profession in this state, and his death though not unexpected will be mourned by many warm friends.

We copy the following from the *Utica Observer* of October 29th:

DANIEL P. BISSELL was born in Randolph, Vermont, on the 27th of May, 1802. He was one of a family of eight—six sons and two daughters—the children of a revolutionary patriot who entered the Continental Army in '76 and fought during the whole war, gaining the rank of Lieutenant in the regular army. In 1809 the BISSELL family emigrated to Ontario County, in this State, where the Doctor was educated. He graduated at the Yale College Medical School in 1826. Returning home he entered upon the practice of his profession in partnership with his brother, Dr. D. H. BISSELL, at Moscow, Livingston county. During his residence in Moscow he was honored with many places of public trust. In 1846 Dr. BISSELL removed to Utica. He became a partner of Dr. GOODSELL, with whom he remained about three years. After the dissolution of that partnership he opened an office on Fayette street, on the first of May, 1851, since which time he was devoted wholly to the practice of his profession. He received many honors at the hands of his medical brethren. He was President of the Livingston County Medical Society; President of the Oneida County Medical Society; President and Vice-President of the New York State Medical Society; and permanent member of the American Medical Association.

In 1842 he was chosen Canal Commissioner by the Legislature. In 1844 he was elected to the same office by the people, and held it until the 1st of January, 1848.—He was a delegate to the Democratic National Convention in 1860. He was also for many years a manager of the Utica Asylum.

His practice as a physician extended over nearly half a century. During all that time he applied himself with studious fidelity to the elevation of his profession For the past four years his health has been failing. His death, although not unexpected, casts a gloom over the community which knew him so well and respected him so highly.

